# Association between peripheral blood markers and immune-related factors on tumor cells in patients with resected primary lung adenocarcinoma

**DOI:** 10.1371/journal.pone.0217991

**Published:** 2019-06-04

**Authors:** Kazuki Takada, Mototsugu Shimokawa, Kensuke Tanaka, Kenichi Kohashi, Akira Haro, Atsushi Osoegawa, Tetsuzo Tagawa, Koichi Azuma, Isamu Okamoto, Yoshinao Oda, Masaki Mori

**Affiliations:** 1 Department of Surgery and Science, Graduate School of Medical Sciences, Kyushu University, Fukuoka, Japan; 2 Clinical Research Institute, National Kyushu Cancer Center, Fukuoka, Japan; 3 Department of Anatomic Pathology, Graduate School of Medical Sciences, Kyushu University, Fukuoka, Japan; 4 Division of Respirology, Neurology, and Rheumatology, Department of Internal Medicine, Kurume University School of Medicine, Fukuoka, Japan; 5 Research Institute for Diseases of the Chest, Graduate School of Medical Sciences, Kyushu University, Fukuoka, Japan; University of South Alabama Mitchell Cancer Institute, UNITED STATES

## Abstract

We sought to identify peripheral blood markers associated with two immune-related factors—programmed cell death-ligand-2 (PD-L2) and indoleamine 2,3-dioxygenase-1 (IDO1)—that are expressed on tumor cells in primary lung adenocarcinoma (AD) specimens. We randomly selected 448 patients (70%) from 640 consecutive patients with resected stage I–III primary lung AD, who had been treated at that point with surgery alone. Expression of PD-L2 and IDO1 in these patients was assessed by immunohistochemistry, and evaluated with respect to peripheral blood markers measured before surgery, including white blood cells, absolute neutrophil count, absolute lymphocyte count, absolute monocyte count (AMC), absolute eosinophil count (AEC), serum C-reactive protein, and serum lactate dehydrogenase levels. Membrane PD-L2 expression and cytoplasmic IDO1 expression were defined by tumor proportion score (TPS); samples with TPS < 1% were considered negative. Logistic regression models were used to identify variables associated with the immune-related factors. Advanced stage (*P* = 0.0090), higher AMC (*P* = 0.0195), and higher AEC (*P* = 0.0015) were independent predictors of IDO1 expression. PD-L2 expression was not associated with any tested peripheral blood markers. Peripheral blood markers, especially AMC and AEC, could potential predict IDO1 expression in lung AD. This study should be replicated in another cohort; further efforts to explore other biomarkers that predict PD-L2 or IDO1 expression are also warranted.

## Introduction

Immune checkpoint inhibitors that target the programmed cell death-1 (PD-1)/programmed cell death-ligand-1 (PD-L1) pathway have been shown to provide survival benefit in non-small cell lung cancer (NSCLC) compared with conventional standard therapy [1–6], and have become a standard treatment option for advanced-stage NSCLC. Moreover, many combination therapies that include such immune checkpoint inhibitors, chemo- or radiation therapy, and another immunotherapy agent (such as an indoleamine 2,3-dioxygenase-1 [IDO1] inhibitor), have been explored to treat various solid tumors, including advanced-stage NSCLC [7–14].

Although the US Food and Drug Administration has approved these immunotherapies only for advanced NSCLC, several ongoing clinical trials for preoperative immunotherapy for stage I–III NSCLC, including CheckMate-816 (ClinicalTrials.gov NCT02998528) and KEYNOTE-091 (ClinicalTrials.gov NCT02504372), offer immunotherapy as a perioperative treatment option for early-stage NSCLC. Moreover, our recent reports indicated that not only PD-L1 but also programmed cell death-ligand-2 (PD-L2), another PD-1 ligand, and IDO1 independently contribute to poor prognoses in patients with resected lung adenocarcinoma (AD) [15, 16]. Therefore, determining the clinical significance of tumor-cell expression of these immune-related factors, PD-L1, PD-L2, and IDO1, and exploring their predictive value in resected NSCLC specimens, could be clinically useful.

Many convenient blood-based immune biomarkers, such as plasma PD-L1, neutrophil-to-lymphocyte ratio, platelet-to-lymphocyte ratio, and C-reactive protein (CRP), have been widely studied as markers for prognosis and monitoring [17–20]. Moreover, Tanizaki et al. showed that a baseline signature of a low absolute neutrophil count (ANC), high absolute lymphocyte count (ALC), and high absolute eosinophil count (AEC) was associated with better outcome for patients treated with nivolumab [21]. Therefore, levels of peripheral blood markers, such as white blood cells (WBC), ANC, ALC, absolute monocyte count (AMC), AEC, serum CRP, and serum lactate dehydrogenase (LDH) levels, could plausibly predict expression of immune-related factors. Our previous report demonstrated that serum CRP was significantly associated with PD-L1 expression and was an independent predictor of PD-L1 expression in NSCLC patients [22]. However, associations among PD-L2 and IDO1 expression and peripheral blood markers in NSCLC patients have not previously been reported.

In this translational study, we examined associations between expression of the immune-related factors, PD-L2 and IDO1, and peripheral blood markers, including WBC, ANC, ALC, AMC, AEC, serum CRP, and serum LDH levels, in patients with resected primary lung AD.

## Materials and methods

### Patients and samples

We retrospectively identified consecutive patients with stage I–III primary lung AD who underwent complete tumor resections between January 2003 and December 2015 at the Department of Surgery and Science, Graduate School of Medical Sciences, Kyushu University. We excluded patients who received neoadjuvant therapy from this study because the possibility of inconsistent tumor microenvironments before and after neoadjuvant therapy could not be ruled out. Finally, we identified 640 patients, of whom 448 (70%) were drawn by simple random sampling using JMP 13.0 (SAS Institute, Cary, NC), to reduce possible bias caused by the retrospective nature of this study, and enrolled them in this single-institution retrospective study.

Preoperative clinical features, including age at surgery, sex, smoking status, clinical tumor-node-metastasis stage (7^th^ edition) [23], and peripheral blood markers (WBC, ANC, ALC, AMC, AEC, serum CRP, and serum LDH) were determined on hospital admission, before surgery. Clinical information was obtained from patients’ medical records; the patients’ information was anonymized before we gained access to it. This study was approved by our institutional review board (Kyushu University, IRB No. 30–402). Our institutional review board waived consent, in view of the retrospective nature of this study.

### PD-L2 and IDO1 expression analysis

We used 4-μm-thick formalin-fixed and paraffin-embedded (FFPE) tumor tissue sections from 640 consecutive patients with primary lung AD to conduct immunohistochemistry tests for IDO1, among which samples from 427 patients were immunohistochemically tested for PD-L2, using commercially available antibodies, as previously described (anti-PD-L2 antibody at 1:200 dilution [mouse monoclonal, clone 176611, R&D Systems, Minneapolis, MN] and anti-IDO1 antibody at 1:200 dilution [mouse monoclonal, clone UMAB126, Origene Technologies, Rockville, MD]) [15, 16, 24–29]. The cohort of 448 patients included 294 patient samples (65.6%) for which PD-L2 immunohistochemistry data were available; these 294 patients had both PD-L2 and IDO1 immunohistochemistry data.

**Fig 1:**
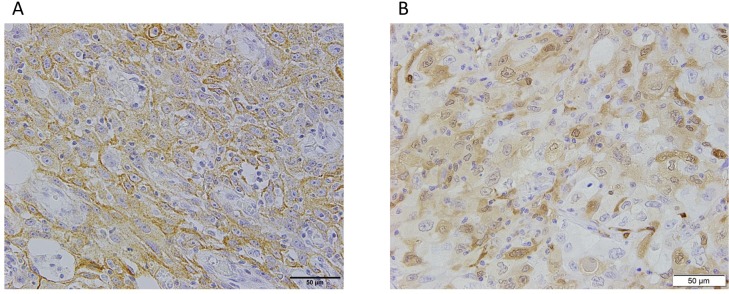
Representative images of immunohistochemical staining for PD-L2 and IDO1 in specimens from patients with primary lung adenocarcinoma. (A) Positive membrane staining for PD-L2. (B) Positive cytoplasmic staining for IDO1. Scale bar: 50 μm. PD-L2, programmed cell death-ligand-2; IDO1, indoleamine 2,3-dioxygenase-1.

All immunohistochemical data were evaluated by two experienced observers (K.T. and K.K.) who were blinded to patients’ clinical status. Final assessments were achieved by consensus. Membrane PD-L2 expression and cytoplasmic IDO1 expression on tumor cells were defined by tumor proportion score (TPS). Samples with TPS < 1% were considered negative in this study, with reference to our previous reports [15, 16]. Representative images of immunohistochemical staining for PD-L2 and IDO1 are shown in **Fig 1**. Although FFPE samples were produced over an approximately 13-year period, the conditions for preparing them remained the same. For example, formalin penetration time has been within 48 hours over this period. Immunohistochemistry stainability did not differ between old (2003–2007) and new (2008–2015) FFPE samples (**S1 Table**).

### Statistical analysis

Patient demographics and baseline characteristics were summarized using descriptive statistics or contingency tables. Associations between immune-related factors expressed on tumor cells and peripheral blood markers were evaluated using Student’s *t*-test. Cut-off values for peripheral blood markers were determined by receiver operating characteristic (ROC) curve analyses. The relationships between PD-L2 and IDO1 expression and other patient characteristics were analyzed by logistic regression model with the backward elimination method. All statistical analyses were performed using JMP 13.0 (SAS Institute, Cary, NC). *P* <0.05 was considered significant.

## Results

### Patient characteristics

Patients’ characteristics are listed in **Table 1**. Their median age was 69 years (range: 34–87 years); 218 (48.7%) were men; 215 (48.0%) were smokers. Disease stages among the 448 patients were stage I: *n* = 344 (76.8%), stage II: *n* = 56 (12.5%), and stage III: *n* = 48 (10.7%).

**Table 1:** Patient demographics and baseline characteristics.

		Value or no. of patients	
Characteristics		All patients (*N* = 448)	Original cohort (*N* = 640)
Age (years)	Median	69	69
	Range	34–87	29–88
Sex	Female	230 (51.3%)	325 (50.8%)
	Male	218 (48.7%)	315 (49.2%)
Smoking status	Never-smoker	233 (52.0%)	326 (50.9%)
	Smoker	215 (48.0%)	314 (49.1%)
Stage	I	344 (76.8%)	489 (76.4%)
	II	56 (12.5%)	78 (12.2%)
	III	48 (10.7%)	73 (11.4%)
WBC (/μL)	Median (average)	5655 (5923)	5660 (5925)
	Range	1620–17150	1620–17150
ANC (/μL)	Median (average)	3396 (3723)	3379 (3687)
	Range	729–14766	729–14766
ALC (/μL)	Median (average)	1571 (1640)	1597 (1676)
	Range	520–5653	450–7832
AMC (/μL)	Median (average)	301 (325)	298 (324)
	Range	81–786	81–807
AEC (/μL)	Median (average)	132 (169)	132 (170)
	Range	0–1339	0–2403
Serum CRP (mg/dL)	Median (average)	0.06 (0.26)	0.06 (0.26)
	Range	0.01–10.73	0.01–10.73
Serum LDH (U/L)	Median (average)	191 (196)	192 (196)
	Range	85–413	75–413
PD-L2*	Negative	91 (31.0%)	123 (28.8%)
	Positive	203 (69.0%)	304 (71.2%)
IDO1	Negative	150 (33.5%)	220 (34.4%)
	Positive	298 (66.5%)	420 (65.6%)

*Cases for which data were available.

AEC: absolute eosinophil count; ALC: absolute lymphocyte count; AMC: absolute monocyte count; ANC: absolute neutrophil count; CRP: C-reactive protein; IDO1: indoleamine 2,3-dioxygenase-1; LDH: lactate dehydrogenase; PD-L2: programmed cell death-ligand-2; WBC: white blood cells.

Among the 448 patients’ samples, 298 (66.5%) were positive for IDO1 expression. Among 294 patients’ samples, 203 (69.0%) were positive for PD-L2 expression. Mean values for laboratory and blood tests were WBC: 5923/μL (range: 1620–17150/μL), ANC: 3723/μL (range: 729–14766/μL), ALC: 1640/μL (range: 520–5653/μL), AMC: 325/μL (range: 81–786/μL), AEC: 169/μL (range: 0–1339/μL), serum CRP: 0.26 mg/dL (range: 0.01–10.73 mg/dL), and serum LDH: 196 U/L (range: 85–413 U/L).

### Association between peripheral blood markers and immune-related factors expressed on tumor cells

First, we examined associations between levels of PD-L2 and IDO1 and peripheral blood markers, including WBC, ANC, ALC, AMC, AEC, serum CRP, and serum LDH (**Table 2**). Among patients with IDO1^+^ tumors, levels of AMC (*P* = 0.0071) and AEC (*P* = 0.0321) were significantly higher than those with IDO1^−^ tumors (Student’s *t*-test). However, PD-L2 expression was not associated with any peripheral blood markers.

**Table 2:** Summary of associations between immune-related factors expressed on tumor cells and peripheral blood markers in patients with resected primary lung adenocarcinoma.

Peripheral blood markers	Peripheral blood markers by immune-related factors, mean value (range)					
	PD-L2, N (%)*		*P*	IDO1, N (%)		*P*
	Negative	Positive		Negative	Positive	
	91 (31.0%)	203 (69.0%)		150 (33.5%)	298 (66.5%)	
WBC (/μL)	5878 (1620–12970)	6027 (2400–17150)	0.5491	5884 (2410–17150)	5942 (1620–14800)	0.7564
ANC (/μL)	3630 (729–9559)	3814 (1041–14766)	0.3790	3770 (1041–14766)	3699 (729–12432)	0.6483
ALC (/μL)	1724 (520–3687)	1686 (549–5653)	0.6224	1610 (520–5653)	1654 (580–3844)	0.4546
AMC (/μL)	319 (81–713)	327 (99–786)	0.5868	303 (99–786)	336 (81–784)	0.0071
AEC (/μL)	174 (0–740)	169 (0–1115)	0.8022	148 (0–652)	180 (0–1339)	0.0321
Serum CRP (mg/dL)	0.21 (0.01–5.29)	0.32 (0.01–10.73)	0.4021	0.16 (0.01–4.50)	0.32 (0.01–10.73)	0.0797
Serum LDH (U/L)	195 (99–343)	197 (113–322)	0.7536	192 (99–309)	198 (85–413)	0.1264

*Cases for which data were available.

AEC: absolute eosinophil count; ALC: absolute lymphocyte count; AMC: absolute monocyte count; ANC: absolute neutrophil count; CRP: C-reactive protein; IDO1: indoleamine 2,3-dioxygenase-1; LDH: lactate dehydrogenase; PD-L2: programmed cell death-ligand-2; WBC: white blood cells.

### Relationships between IDO1 expression and other patient characteristics

**Fig 2:**
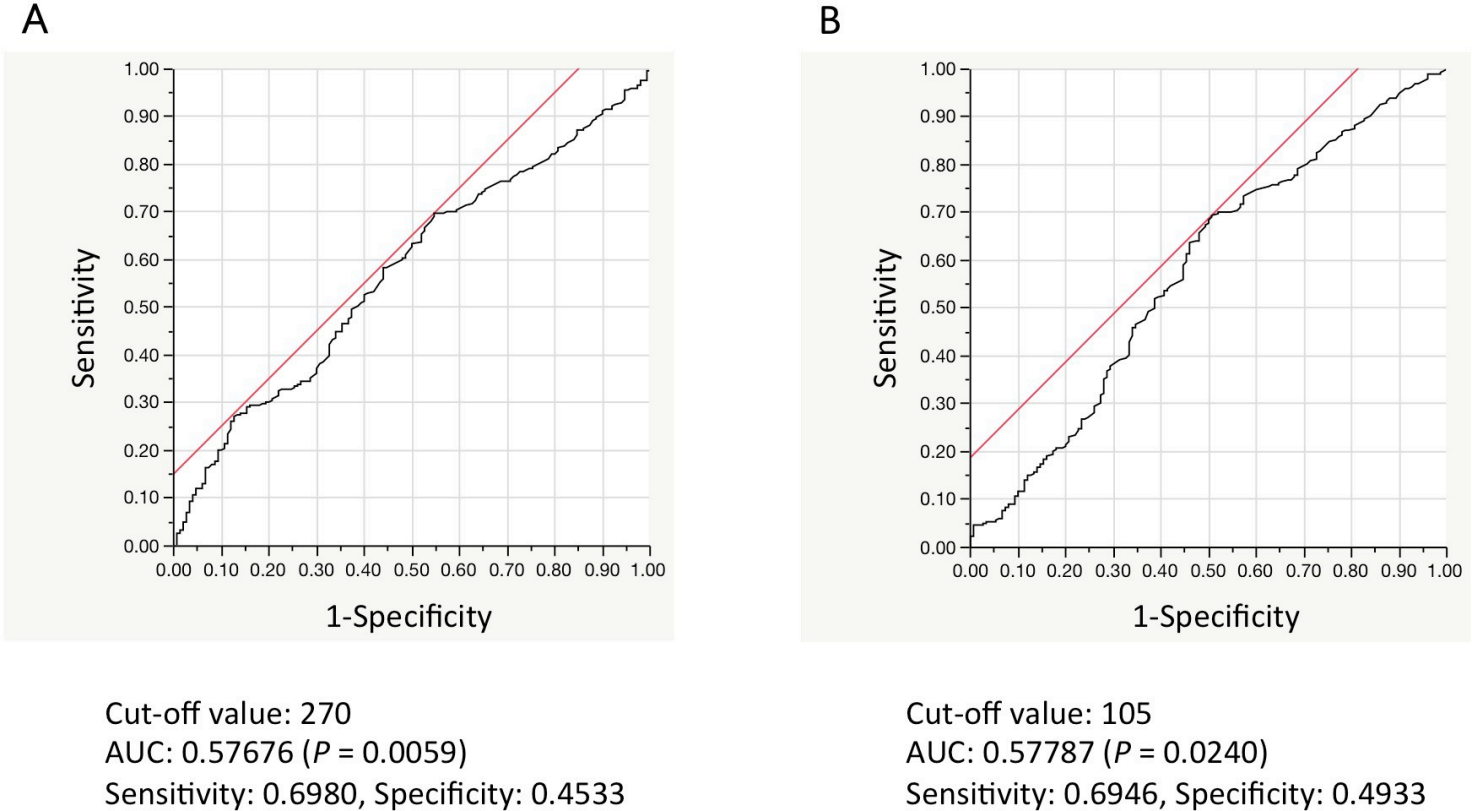
Representative images of receiver operating characteristic curves to determine cut-off values in the analysis of the relationship between IDO1 and patient characteristics. (A) AMC and (B) AEC. AMC: absolute monocyte count; AEC: absolute eosinophil count; IDO1: indoleamine 2,3-dioxygenase-1; AUC: area under curve.

Next, we examined associations between IDO1 expression and other patient characteristics. We determined optimal AMC and AEC cut-off levels using ROC curve analyses (**Fig 2**). In multivariate analyses, advanced stage (*P* = 0.0090), higher AMC (*P* = 0.0195), and higher AEC (*P* = 0.0015) were independent predictors of IDO1 expression (**Table 3**).

**Table 3:** Univariate and multivariate analyses of relationships between IDO1 expression and other patient characteristics.

Characteristics		Univariate analysis		Multivariate analysis	
		OR (95%CI)	*P*	OR (95%CI)	*P*
Age (years)	≥ 70/< 70	1.11 (0.75–1.65)	0.5947		
Sex	Male/female	1.50 (1.01–2.22)	0.0459		
Smoking status	Smoker/never-smoker	1.38 (0.93–2.05)	0.1100		
Stage	≥ II/I	2.06 (1.23–3.44)	0.0057	2.01 (1.19–3.38)	0.0090
AMC (/μL)	≥ 270/< 270	1.92 (1.28–2.88)	0.0017	1.65 (1.08–2.52)	0.0195
AEC (/μL)	≥ 105/< 105	2.21 (1.48–3.32)	0.0001	1.97 (1.30–2.99)	0.0015

AEC: absolute eosinophil count; AMC: absolute monocyte count; CI: confidence interval; IDO1: indoleamine 2,3-dioxygenase-1; OR: odds ratio.

### IDO1 expression rates by clinical characteristics

Based on the above results, we analyzed percentages of IDO1^+^ tumors by clinical characteristics (**Table 4**). Among patients with higher AMC, higher AEC and advanced stage disease, 84.3% (43/51) had IDO1^+^ tumors, compared with 42.4% (28/66) among patients with lower AMC, lower AEC and stage I disease.

**Table 4:** Summary of IDO1 expression frequency by clinical characteristics.

AMC (/μL)	AEC (/μL)	Percentage of IDO1+ tumors		
		Stage I	Stage II/III	Overall
< 270	< 105	42.4% (28/66)	66.7% (10/15)	46.9% (38/81)
< 270	≥ 105	63.8% (37/58)	78.9% (15/19)	67.5% (52/77)
≥ 270	< 105	61.5% (40/65)	68.4% (13/19)	63.1% (53/84)
≥ 270	≥ 105	72.3% (112/155)	84.3% (43/51)	75.2% (155/206)

AEC: absolute eosinophil count; AMC: absolute monocyte count; IDO1: indoleamine 2,3-dioxygenase-1.

## Discussion

In the current study, we examined associations between the expression levels of two immune-related factors (PD-L2 and IDO1) and peripheral blood markers (WBC, ANC, ALC, AMC, AEC, serum CRP, and serum LDH) in patients who had undergone resections of primary lung AD. We found that advanced stage, higher AMC, and higher AEC were independent predictors of IDO1 expression. However, PD-L2 expression was not associated with any tested peripheral blood markers. Therefore, peripheral blood markers such as AMC and AEC could plausibly be used to predict expression of the immune-related factor IDO1 in lung AD.

IDO1 is a rate-limiting enzyme that catabolizes conversion of tryptophan into a stable metabolite under the kynurenine pathway. It is expressed by antigen-presenting cells, including tumor cells that are exposed to interferon-γ and other proinflammatory stimuli in the tumor microenvironment [30, 31]. IDO1 plays an important role in immune tolerance [32, 33], and is a potential immune-based therapeutic target. In particular, a combination of an IDO1 inhibitor and a PD-1/PD-L1 inhibitor is expected to be an effective treatment option that is currently being tested in many ongoing clinical trials for patients with various solid tumors, including NSCLC [13, 14, 34–39]. We therefore believe that identifying predictive markers for IDO1 expression is important. We recently evaluated relationships among IDO1 expression, patient prognosis and clinicopathological features (including PD-L1 expression) in lung AD [16]. In that study, tumor grade, vascular invasion, and PD-L1 expression were independent predictors of IDO1 expression, but these were all pathological findings rather than clinical features. In that context, positive correlations between IDO1 expression and higher AMC and higher AEC are noteworthy. Moreover, our results imply the existence of a mechanistic link between IDO1 expression and higher AMC and higher AEC. Recently, Tanizaki et al. showed that high AEC was significantly associated with better outcomes among patients with NSCLC treated with nivolumab [21], and Seeber et al. found that high IDO1 expression was associated with response to anti-PD-1 drugs in metastatic renal cell carcinoma [40]. Together, these findings suggest that IDO1 expression could also be a predictor for response to PD-1/PD-L1 immune checkpoint inhibitors in NSCLC. Factors to predict IDO1 expression may therefore be clinically useful. Future studies of the relationship between IDO1 expression and response to anti-PD-1/PD-L1 inhibitors in NSCLC are required.

PD-L2 is the second identified ligand for the PD-1 [41]. Few studies have explored PD-L2 expression in NSCLC, and the role of PD-L2 in the tumor microenvironment; a prognostic or predictive marker has not been established [42–46]. However, our previous report showed that PD-L2 expression might be a target of immune checkpoint inhibitors in NSCLC patients with negative PD-L1 expression [25]. Therefore, we thought that determining the clinical significance of PD-L2 expression in NSCLC was important. Recently, we examined the prognostic effect of PD-L2 expression on NSCLC patients after surgery [15, 29], and the radiological features of PD-L2-positive lung AD [28].

The present study is the first to examine the association between PD-L2 expression and peripheral blood markers. Notably, PD-L2 expression was not associated with any peripheral blood markers. Further studies of the clinical roles of PD-L2 are therefore required in the future.

PD-L1 is a key factor in cancer immunotherapy, and subset analyses in many clinical trials—including the CheckMate, KEYNOTE, POPLAR, and OAK studies—showed a close correlation between PD-L1 expression and the efficacy of anti-PD-1/PD-L1 inhibitors [1, 3–6, 47]. Therefore, identifying predictive markers for PD-L1 expression is important. We have examined the association between PD-L1 expression and both clinical factors and characteristics of imaging modalities such as computed tomography (CT) and ^18^F-fluorodeoxyglucose positron emission tomography/CT [48–50]. Our previous report already showed that serum CRP was significantly associated with PD-L1 expression in different statistical analyses, and elevated serum CRP may represent both the host’s chronic inflammatory status, and the host’s immune response to the tumor, through elevation of inflammatory cytokines [22]. From these studies, we could predict PD-L1 expression by using clinical features, imaging modality characteristics, and peripheral blood markers.

This study had several limitations. First, because it was a single-institution retrospective study and not a trial-based correlative study, the potential for bias cannot be excluded. Second, immunohistochemical analyses for PD-L2 and IDO1 were conducted using only the clones 176611 and UMAB126, respectively, which have not been evaluated in a clinical setting. Therefore, these expressions should be further evaluated using other antibodies. The third limitation was that we examined associations between levels of immune-related factors and peripheral blood markers only in lung AD. We also should conduct the same analyses in other histological types of lung cancer such as squamous cell carcinoma. Fourth, the area under the ROC curves for IDO1 and AMC and AEC seems to be too low to be clinically useful. Therefore, the current findings wouldn’t drive testing or serve as pilot data for larger validation studies.

In conclusion, peripheral blood markers could be potential biomarkers to predict the expression of IDO1 in lung adenocarcinoma. However, the results of this study cannot directly serve as pilot data for a larger validation study. We should conduct the same study in another (possibly multicenter) cohort; prospective studies may also be warranted. Further efforts to explore other predictive biomarkers for PD-L2 and IDO1 expression could be useful and interesting.

## Supporting information

S1 Table The immunohistochemical stainability of IDO1 antibody between old (2003–2007) and new (2008–2015) formalin-fixed and paraffin-embedded samples. The stainability did not differ between old (2003–2007) and new (2008–2015) formalin-fixed and paraffin-embedded samples in (A) 640 and (B) 448 patients. IDO1: indoleamine 2,3-dioxygenase-1.
(DOCX)

## References

[1] Rittmeyer A, Barlesi F, Waterkamp D, Park K, Ciardiello F, von Pawel J, et al Atezolizumab versus docetaxel in patients with previously treated non-small-cell lung cancer (OAK): a phase 3, open-label, multicentre randomised controlled trial. Lancet (London, England). 2016 10.1016/s0140-6736(16)32517-x . 27979383 PMC6886121

[2] Reck M, Rodriguez-Abreu D, Robinson AG, Hui R, Csoszi T, Fulop A, et al Pembrolizumab versus Chemotherapy for PD-L1-Positive Non-Small-Cell Lung Cancer. The New England journal of medicine. 2016 10.1056/NEJMoa1606774 . 27718847

[3] Herbst RS, Baas P, Kim DW, Felip E, Perez-Gracia JL, Han JY, et al Pembrolizumab versus docetaxel for previously treated, PD-L1-positive, advanced non-small-cell lung cancer (KEYNOTE-010): a randomised controlled trial. Lancet (London, England). 2016;387(10027):1540–50. WOS:000373741600030. 10.1016/S0140-6736(15)01281-7 26712084

[4] Fehrenbacher L, Spira A, Ballinger M, Kowanetz M, Vansteenkiste J, Mazieres J, et al Atezolizumab versus docetaxel for patients with previously treated non-small-cell lung cancer (POPLAR): a multicentre, open-label, phase 2 randomised controlled trial. Lancet (London, England). 2016;387(10030):1837–46. WOS:000375056100032. 10.1016/S0140-6736(16)00587-0 26970723

[5] Brahmer J, Reckamp KL, Baas P, Crino L, Eberhardt WE, Poddubskaya E, et al Nivolumab versus Docetaxel in Advanced Squamous-Cell Non-Small-Cell Lung Cancer. The New England journal of medicine. 2015;373(2):123–35. Epub 2015/06/02. 10.1056/NEJMoa1504627 . 26028407 PMC4681400

[6] Borghaei H, Paz-Ares L, Horn L, Spigel DR, Steins M, Ready NE, et al Nivolumab versus Docetaxel in Advanced Nonsquamous Non-Small-Cell Lung Cancer. The New England journal of medicine. 2015;373(17):1627–39. Epub 2015/09/29. 10.1056/NEJMoa1507643 . 26412456 PMC5705936

[7] Takamori S, Toyokawa G, Takada K, Shoji F, Okamoto T, Maehara Y. Combination Therapy of Radiotherapy and Anti-PD-1/PD-L1 Treatment in Non-Small-cell Lung Cancer: A Mini-review. Clinical lung cancer. 2018;19(1):12–6. Epub 2017/07/26. 10.1016/j.cllc.2017.06.015 . 28739315

[8] Socinski MA, Jotte RM, Cappuzzo F, Orlandi F, Stroyakovskiy D, Nogami N, et al Atezolizumab for First-Line Treatment of Metastatic Nonsquamous NSCLC. The New England journal of medicine. 2018;378(24):2288–301. Epub 2018/06/05. 10.1056/NEJMoa1716948 . 29863955

[9] Paz-Ares L, Luft A, Tafreshi A, Gumus M, Mazieres J, Hermes B, et al Phase 3 study of carboplatin-paclitaxel/nab-paclitaxel (Chemo) with or without pembrolizumab (Pembro) for patients (Pts) with metastatic squamous (Sq) non-small cell lung cancer (NSCLC). Journal of clinical oncology: official journal of the American Society of Clinical Oncology. 2018;36(suppl; abstr 105):suppl; abstr 105. 10.1200/JCO.19.01348 31751163

[10] Jotte RM, Cappuzzo F, Vynnychenko I, Stroyakovskiy D, Abreu DR, Hussein MA, et al IMpower131: Primary PFS and safety analysis of a randomized phase III study of atezolizumab + carboplatin + paclitaxel or nab-paclitaxel vs carboplatin + nab-paclitaxel as 1L therapy in advanced squamous NSCLC. Journal of clinical oncology: official journal of the American Society of Clinical Oncology. 2018;36(suppl; abstr LBA9000):suppl; abstr LBA9000.

[11] Hellmann MD, Ciuleanu TE, Pluzanski A, Lee JS, Otterson GA, Audigier-Valette C, et al Nivolumab plus Ipilimumab in Lung Cancer with a High Tumor Mutational Burden. The New England journal of medicine. 2018;378(22):2093–104. Epub 2018/04/17. 10.1056/NEJMoa1801946 . 29658845 PMC7193684

[12] Gandhi L, Rodriguez-Abreu D, Gadgeel S, Esteban E, Felip E, De Angelis F, et al Pembrolizumab plus Chemotherapy in Metastatic Non-Small-Cell Lung Cancer. The New England journal of medicine. 2018;378(22):2078–92. Epub 2018/04/17. 10.1056/NEJMoa1801005 . 29658856

[13] Hui R, Munteanu M, Zhao Y, Luo Y, Samkari A, Garassino MC. ECHO-306/KEYNOTE-715: A phase 3 study of first-line epacadostat plus pembrolizumab with or without platinum-based chemotherapy vs pembrolizumab plus platinum-based chemotherapy plus placebo for metastatic non–small cell lung cancer (mNSCLC). Journal of clinical oncology: official journal of the American Society of Clinical Oncology. 2018;36(suppl; abstr TPS9104):suppl; abstr TPS9104.

[14] Awad MM, Munteanu M, Zhao Y, Xu L, Samkari A, Paz-Ares L. ECHO-305/KEYNOTE-654: A phase 3, randomized, double-blind study of first-line epacadostat plus pembrolizumab vs pembrolizumab plus placebo for metastatic non–small cell lung cancer (mNSCLC) with high PD-L1 levels. Journal of clinical oncology: official journal of the American Society of Clinical Oncology. 2018;36(suppl; abstr TPS9109):suppl; abstr TPS9109.

[15] Takamori S, Takada K, Azuma K, Jogo T, Shimokawa M, Toyokawa G, et al Prognostic Impact of Programmed Death-Ligand 2 Expression in Primary Lung Adenocarcinoma Patients. Annals of surgical oncology. 2019 Epub 2019/03/01. 10.1245/s10434-019-07231-z . 30815801

[16] Kozuma Y, Takada K, Toyokawa G, Kohashi K, Shimokawa M, Hirai F, et al Indoleamine 2,3-dioxygenase 1 and programmed cell death-ligand 1 co-expression correlates with aggressive features in lung adenocarcinoma. European journal of cancer (Oxford, England: 1990). 2018;101:20–9. Epub 2018/07/18. 10.1016/j.ejca.2018.06.020 . 30014971

[17] Bagley SJ, Kothari S, Aggarwal C, Bauml JM, Alley EW, Evans TL, et al Pretreatment neutrophil-to-lymphocyte ratio as a marker of outcomes in nivolumab-treated patients with advanced non-small-cell lung cancer. Lung cancer (Amsterdam, Netherlands). 2017;106:1–7. Epub 2017/03/14. 10.1016/j.lungcan.2017.01.013 . 28285682

[18] Okuma Y, Hosomi Y, Nakahara Y, Watanabe K, Sagawa Y, Homma S. High plasma levels of soluble programmed cell death ligand 1 are prognostic for reduced survival in advanced lung cancer. Lung cancer (Amsterdam, Netherlands). 2017;104:1–6. Epub 2017/02/19. 10.1016/j.lungcan.2016.11.023 . 28212990

[19] Brustugun OT, Sprauten M, Helland A. C-reactive protein (CRP) as a predictive marker for immunotherapy in lung cancer. Journal of clinical oncology: official journal of the American Society of Clinical Oncology. 2016.

[20] Diem S, Schmid S, Krapf M, Flatz L, Born D, Jochum W, et al Neutrophil-to-Lymphocyte ratio (NLR) and Platelet-to-Lymphocyte ratio (PLR) as prognostic markers in patients with non-small cell lung cancer (NSCLC) treated with nivolumab. Lung cancer (Amsterdam, Netherlands). 2017;111:176–81. Epub 2017/08/26. 10.1016/j.lungcan.2017.07.024 . 28838390

[21] Tanizaki J, Haratani K, Hayashi H, Chiba Y, Nakamura Y, Yonesaka K, et al Peripheral Blood Biomarkers Associated with Clinical Outcome in Non-Small Cell Lung Cancer Patients Treated with Nivolumab. Journal of thoracic oncology: official publication of the International Association for the Study of Lung Cancer. 2018;13(1):97–105. Epub 2017/11/25. 10.1016/j.jtho.2017.10.030 . 29170120

[22] Akamine T, Takada K, Toyokawa G, Kinoshita F, Matsubara T, Kozuma Y, et al Association of preoperative serum CRP with PD-L1 expression in 508 patients with non-small cell lung cancer: A comprehensive analysis of systemic inflammatory markers. Surgical oncology. 2018;27(1):88–94. Epub 2018/03/20. 10.1016/j.suronc.2018.01.002 . 29549910

[23] Goldstraw P, Crowley J, Chansky K, Giroux DJ, Groome PA, Rami-Porta R, et al The IASLC Lung Cancer Staging Project: proposals for the revision of the TNM stage groupings in the forthcoming (seventh) edition of the TNM Classification of malignant tumours. Journal of thoracic oncology: official publication of the International Association for the Study of Lung Cancer. 2007;2(8):706–14. Epub 2007/09/01. 10.1097/JTO.0b013e31812f3c1a . 17762336

[24] Takada K, Kohashi K, Shimokawa M, Haro A, Osoegawa A, Tagawa T, et al Co-expression of IDO1 and PD-L1 in lung squamous cell carcinoma: Potential targets of novel combination therapy. Lung cancer (Amsterdam, Netherlands). 2019;128:26–32. Epub 2019/01/16. 10.1016/j.lungcan.2018.12.008 . 30642449

[25] Takamori S, Takada K, Toyokawa G, Azuma K, Shimokawa M, Jogo T, et al PD-L2 Expression as a Potential Predictive Biomarker for the Response to Anti-PD-1 Drugs in Patients with Non-small Cell Lung Cancer. Anticancer research. 2018;38(10):5897–901. Epub 2018/10/03. 10.21873/anticanres.12933 . 30275216

[26] Takamori S, Takada K, Azuma K, Jogo Y, Kinoshita F, Kozuma Y, et al Prognostic Impact of PD-L2 Expression and Association with PD-L1 in Patients with Small-cell Lung Cancer. Anticancer research. 2018;38(10):5903–7. Epub 2018/10/03. 10.21873/anticanres.12934 . 30275217

[27] Takada K, Toyokawa G, Tagawa T, Shimokawa M, Kohashi K, Haro A, et al Radiological Features of IDO1(+)/PDL1(+) Lung Adenocarcinoma: A Retrospective Single-institution Study. Anticancer research. 2018;38(9):5295–303. Epub 2018/09/09. 10.21873/anticanres.12856 . 30194181

[28] Takada K, Toyokawa G, Azuma K, Takamori S, Jogo T, Hirai F, et al Radiological Features of Programmed Cell Death-Ligand 2-positive Lung Adenocarcinoma: A Single-institution Retrospective Study. In vivo (Athens, Greece). 2018;32(6):1541–50. Epub 2018/10/24. 10.21873/invivo.11412 . 30348714 PMC6365729

[29] Matsubara T, Takada K, Azuma K, Takamori S, Toyokawa G, Haro A, et al A Clinicopathological and Prognostic Analysis of PD-L2 Expression in Surgically Resected Primary Lung Squamous Cell Carcinoma. Annals of surgical oncology. 2019 Epub 2019/03/01. 10.1245/s10434-019-07257-3 . 30815803

[30] Zhai L, Spranger S, Binder DC, Gritsina G, Lauing KL, Giles FJ, et al Molecular Pathways: Targeting IDO1 and Other Tryptophan Dioxygenases for Cancer Immunotherapy. Clinical cancer research: an official journal of the American Association for Cancer Research. 2015;21(24):5427–33. Epub 2015/11/01. 10.1158/1078-0432.ccr-15-0420 26519060 PMC4681601

[31] Theate I, van Baren N, Pilotte L, Moulin P, Larrieu P, Renauld JC, et al Extensive profiling of the expression of the indoleamine 2,3-dioxygenase 1 protein in normal and tumoral human tissues. Cancer immunology research. 2015;3(2):161–72. Epub 2014/10/02. 10.1158/2326-6066.CIR-14-0137 . 25271151

[32] Della Chiesa M, Carlomagno S, Frumento G, Balsamo M, Cantoni C, Conte R, et al The tryptophan catabolite L-kynurenine inhibits the surface expression of NKp46- and NKG2D-activating receptors and regulates NK-cell function. Blood. 2006;108(13):4118–25. Epub 2006/08/12. 10.1182/blood-2006-03-006700 . 16902152

[33] Fallarino F, Grohmann U, You S, McGrath BC, Cavener DR, Vacca C, et al The combined effects of tryptophan starvation and tryptophan catabolites down-regulate T cell receptor zeta-chain and induce a regulatory phenotype in naive T cells. Journal of immunology (Baltimore, Md: 1950). 2006;176(11):6752–61. Epub 2006/05/20. 10.4049/jimmunol.176.11.6752 . 16709834

[34] Powles T, Bellmunt J, Petrylak DP, Fong L, Nishiyama H, Sternberg CN, et al Pembrolizumab (pembro) plus epacadostat or placebo for locally advanced or metastatic urothelial carcinoma (UC) after failure of first-line platinum-containing chemotherapy: KEYNOTE-698/ECHO-303. Journal of clinical oncology: official journal of the American Society of Clinical Oncology. 2018;36(suppl; abstr TPS4586):suppl; abstr TPS4586.

[35] Balar AV, Plimack ER, Grivas P, Necchi A, Santis MD, Pang L, et al Phase 3, randomized, double-blind trial of pembrolizumab plus epacadostat or placebo for cisplatin-ineligible urothelial carcinoma (UC): KEYNOTE-672/ECHO-307. Journal of clinical oncology: official journal of the American Society of Clinical Oncology. 2018;36(suppl; abstr TPS4587):suppl; abstr TPS4587.

[36] Cohen EEW, Mehra R, Psyrri A, Bauman JE, Schaub R, Zhou L, et al ECHO-310: A phase 3, randomized trial of epacadostat + nivolumab + chemo vs EXTREME as first-line treatment of recurrent/metastatic SCCHN. Journal of clinical oncology: official journal of the American Society of Clinical Oncology. 2018;36(suppl; abstr TPS6092):suppl; abstr TPS6092.

[37] Cohen EEW, Rischin D, Pfister DG, Vermorken JB, Zhao Y, Gowda H, et al A phase 3, randomized, open-label study of epacadostat plus pembrolizumab, pembrolizumab monotherapy, and the EXTREME regimen as first-line treatment for recurrent/metastatic head and neck squamous cell carcinoma (R/M SCCHN): ECHO-304/KEYNOTE-669. Journal of clinical oncology: official journal of the American Society of Clinical Oncology. 2018;36(suppl; abstr TPS6090):suppl; abstr TPS6090.

[38] Daud A, Saleh MN, Hu J, Bleeker JS, Riese MJ, Meier R, et al Epacadostat plus nivolumab for advanced melanoma: Updated phase 2 results of the ECHO-204 study. Journal of clinical oncology: official journal of the American Society of Clinical Oncology. 2018;36(suppl; abstr 9511):suppl; abstr 9511.

[39] Long GV, Dummer R, Hamid O, Gajewski T, Caglevic C, Dalle S, et al Epacadostat (E) plus pembrolizumab (P) versus pembrolizumab alone in patients (pts) with unresectable or metastatic melanoma: Results of the phase 3 ECHO-301/KEYNOTE-252 study. Journal of clinical oncology: official journal of the American Society of Clinical Oncology. 2018;36(suppl; abstr 108):suppl; abstr 108.

[40] Seeber A, Klinglmair G, Fritz J, Steinkohl F, Zimmer KC, Aigner F, et al High IDO-1 expression in tumor endothelial cells is associated with response to immunotherapy in metastatic renal cell carcinoma. Cancer science. 2018;109(5):1583–91. Epub 2018/03/03. 10.1111/cas.13560 29498788 PMC5980224

[41] Latchman Y, Wood CR, Chernova T, Chaudhary D, Borde M, Chernova I, et al PD-L2 is a second ligand for PD-1 and inhibits T cell activation. Nature immunology. 2001;2(3):261–8. Epub 2001/02/27. 10.1038/85330 . 11224527

[42] Koh J, Go H, Keam B, Kim MY, Nam SJ, Kim TM, et al Clinicopathologic analysis of programmed cell death-1 and programmed cell death-ligand 1 and 2 expressions in pulmonary adenocarcinoma: comparison with histology and driver oncogenic alteration status. Modern pathology: an official journal of the United States and Canadian Academy of Pathology, Inc. 2015;28(9):1154–66. Epub 2015/07/18. 10.1038/modpathol.2015.63 . 26183759

[43] Kim MY, Koh J, Kim S, Go H, Jeon YK, Chung DH. Clinicopathological analysis of PD-L1 and PD-L2 expression in pulmonary squamous cell carcinoma: Comparison with tumor-infiltrating T cells and the status of oncogenic drivers. Lung cancer (Amsterdam, Netherlands). 2015;88(1):24–33. Epub 2015/02/11. 10.1016/j.lungcan.2015.01.016 . 25662388

[44] Calles A, Liao X, Sholl LM, Rodig SJ, Freeman GJ, Butaney M, et al Expression of PD-1 and Its Ligands, PD-L1 and PD-L2, in Smokers and Never Smokers with KRAS-Mutant Lung Cancer. Journal of thoracic oncology: official publication of the International Association for the Study of Lung Cancer. 2015;10(12):1726–35. Epub 2015/10/17. 10.1097/jto.0000000000000687 . 26473645

[45] Zhang Y, Wang L, Li Y, Pan Y, Wang R, Hu H, et al Protein expression of programmed death 1 ligand 1 and ligand 2 independently predict poor prognosis in surgically resected lung adenocarcinoma. OncoTargets and therapy. 2014;7:567–73. Epub 2014/04/22. 10.2147/OTT.S59959 24748806 PMC3990506

[46] Konishi J, Yamazaki K, Azuma M, Kinoshita I, Dosaka-Akita H, Nishimura M. B7-H1 expression on non-small cell lung cancer cells and its relationship with tumor-infiltrating lymphocytes and their PD-1 expression. Clinical cancer research: an official journal of the American Association for Cancer Research. 2004;10(15):5094–100. Epub 2004/08/07. 10.1158/1078-0432.ccr-04-0428 . 15297412

[47] Reck M, Rodriguez-Abreu D, Robinson AG, Hui R, Csoszi T, Fulop A, et al Pembrolizumab versus Chemotherapy for PD-L1-Positive Non-Small-Cell Lung Cancer. The New England journal of medicine. 2016;375(19):1823–33. Epub 2016/10/11. 10.1056/NEJMoa1606774 . 27718847

[48] Toyokawa G, Takada K, Okamoto T, Shimokawa M, Kozuma Y, Matsubara T, et al Computed Tomography Features of Lung Adenocarcinomas With Programmed Death Ligand 1 Expression. Clinical lung cancer. 2017;18(6):e375–e83. Epub 2017/04/08. 10.1016/j.cllc.2017.03.008 . 28385373

[49] Takada K, Toyokawa G, Okamoto T, Baba S, Kozuma Y, Matsubara T, et al Metabolic characteristics of programmed cell death-ligand 1-expressing lung cancer on 18 F-fluorodeoxyglucose positron emission tomography/computed tomography. Cancer medicine. 2017;6(11):2552–61. Epub 2017/10/06. 10.1002/cam4.1215 . 28980429 PMC5673920

[50] Takada K, Okamoto T, Shoji F, Shimokawa M, Akamine T, Takamori S, et al Clinical Significance of PD-L1 Protein Expression in Surgically Resected Primary Lung Adenocarcinoma. Journal of thoracic oncology: official publication of the International Association for the Study of Lung Cancer. 2016;11(11):1879–90. Epub 2016/10/25. 10.1016/j.jtho.2016.06.006 . 27346415

