# Supplementary material for: Association between peripheral blood markers and immune-related factors on tumor cells in patients with resected primary lung adenocarcinoma
Source: PLoS One. 2019 Jun 4;14(6):e0217991. doi: 10.1371/journal.pone.0217991 (PMC6548429; doi:10.1371/journal.pone.0217991)
Supplement: S1 Table — The stainability did not differ between old (2003–2007) and new (2008–2015) formalin-fixed and paraffin-embedded samples in (A) 640 and (B) 448 patients. IDO1: indoleamine 2,3-dioxygenase-1. (DOCX) [file pone.0217991.s001.docx]

**S1 Table.** The immunohistochemical stainability of IDO1 antibody between old (2003-2007) and new (2008-2015) formalin-fixed and paraffin-embedded samples.

Table A (*N* = 640)

|  |  | ***N* (%)** | **2003-2007** | **2008-2015** | ***P* value** |
| --- | --- | --- | --- | --- | --- |
|  |  |  |  |  |  |
| IDO1 | Negative | 220 (34.4%) | 75 (37.3%) | 145 (33.0%) | 0.3240 |
|  | Positive | 420 (65.6%) | 126 (62.7%) | 294 (67.0%) |  |

IDO1, indoleamine 2,3-dioxygenase 1.

Table B (*N* = 448)

|  |  | ***N* (%)** | **2003-2007** | **2008-2015** | ***P* value** |
| --- | --- | --- | --- | --- | --- |
|  |  |  |  |  |  |
| IDO1 | Negative | 150 (33.5%) | 52 (38.5%) | 98 (31.3%) | 0.1562 |
|  | Positive | 298 (66.5%) | 83 (61.5%) | 215 (68.7%) |  |

IDO1, indoleamine 2,3-dioxygenase 1.
